# Correlation between calf circumference and skeletal mass index among type 2 diabetes mellitus individuals with sarcopenia

**DOI:** 10.1038/s41598-026-59139-w

**Published:** 2026-06-22

**Authors:** Deeksha Shettigar, G. Arun Maiya, Sahana Shetty, K. V. Rajagopal, Rama Bhat, Vasudeva Acharya, Shreemathi S. Mayya, Shetty Shrija Jaya

**Affiliations:** 1 Department of Physiotherapy, Manipal College of Health Professions (MCHP), Manipal Academy of Higher Education, Manipal, India; 2 Department of Endocrinology, Kasturba Medical College, Manipal Academy of Higher Education, Manipal, India; 3 Department of Radiodiagnosis, Kasturba Medical College, Manipal Academy of Higher Education, Manipal, Karnataka India; 4 Department of Medicine, Kasturba Medical College, Manipal Academy of Higher Education, Manipal, India; 5 Department of Data Science, Prasanna School of Public Health, Manipal Academy of Higher Education, Manipal, India

**Keywords:** Diabetes mellitus, Sarcopenia, Muscle mass, Body composition, Bioelectrical impedance analysis, Endocrinology, Health care

## Abstract

Individuals diagnosed with type 2 diabetes mellitus(T2DM) are more likely to develop sarcopenia. Sarcopenia is one of the new complications that lead to decreased muscle strength and mass. The objective of the present study is to evaluate the correlation between calf circumference and skeletal mass index among T2DM participants with sarcopenia. This study included 62 individuals diagnosed with T2DM with Sarcopenia aged between 40 and 70 years. The examiner noted demographic details, including age, sex, height, weight, and BMI. The correlation between skeletal mass index and calf circumference was analyzed using Spearman’s correlation. The mean age of the 29 female participants in this study was 58.2 ± 8.57 years, and the 33 males were 61.1 ± 8.37 years. A significant positive moderate correlation between calf circumference and skeletal mass index was found (Spearman’s *r* = 0.58 for females and 0.62 for males, respectively, *p* < 0.001). The multivariable linear regression model was statistically significant (R² = 0.556, adjusted R² = 0.515, F(5,54) = 13.547, *p* < 0.001). Assessment of calf circumference is a feasible, easy-to-incorporate, and cost-effective way to screen patients with T2DM and sarcopenia, and it can be incorporated into tertiary healthcare settings.

## Introduction

Diabetes mellitus is a group of metabolic disorders resulting from defects in insulin secretion, insulin action, or both, characterized by hyperglycemia^1^. A common chronic consequence in people with T2DM is sarcopenia^2^. The term “Sarcopenia” refers to an age-related, involuntary loss of skeletal muscle mass and strength^3^. The current consensus is that sarcopenia is a brand-new complication of T2DM, which not only lowers quality of life but also raises the risk of physical disability and even mortality^4^. Sarcopenia is linked to several comorbidities, such as a higher risk of falling and a higher prevalence of metabolic illnesses (such as obesity and type 2 diabetes mellitus), in addition to limits in muscle mass/quality and function^5^. The primary markers of T2DM include insulin resistance, inflammation, elevated levels of advanced glycation end products (AGEs), and oxidative stress. These characteristics may accelerate the development of sarcopenia by causing losses in skeletal muscle mass, strength, and function^6^. One of the main contributing reasons to the onset of sarcopenia is considered to be insulin resistance^7^. In diabetic patients, as people age, fat mass increases, anabolic resistance worsens, and lean mass decreases more rapidly than in nondiabetic patients^8^. When comparing patients with T2DM to those with euglycemia, the risk of sarcopenia was 1.5 times higher^9^. Patients with type 2 diabetes had an 18% prevalence of sarcopenia^10^. Globally, the prevalence of sarcopenia was estimated at 10% to 16% in the elderly population^11^.

Sarcopenia affects the musculoskeletal system by decreasing muscle mass and strength, leading to decreased muscle size, increased risk of falls due to poor balance, reduced walking speed, trouble climbing stairs, decreased hand grip strength, frailty, and fractures, which can lead to hospitalization and surgeries^4,9^. In older people, calf circumference has been identified as a sensitive anthropometric measure of muscle mass^12^. This should make it easier to identify the condition early and implement it in public health^13^. It has been recommended that measuring calf circumference is an easy and affordable way to assess an aged person’s muscle mass^14^.

Sarcopenia’s vital component is the assessment of skeletal mass index (SMI)^13^. Sarcopenia can be measured using various methods, including dual-energy x-ray absorptiometry (DEXA), ultrasonography, computed tomography, anthropometry, bioelectric impedance analysis (BIA), and Magnetic Resonance Imaging (MRI)^15^. These methods are expensive and unavailable in all healthcare settings. When predicting sarcopenia, calf circumference has a moderate to high sensitivity and specificity^16^. However, in clinical practice, early screening for sarcopenia using simple, feasible, and cost-effective measures is essential for timely identification and intervention, particularly in resource-limited healthcare settings. Anthropometric measures, such as calf circumference, may serve as practical screening tools for early detection of sarcopenia among older adults and individuals at risk. Therefore, the objective of the present study was to determine the correlation between skeletal mass index and calf circumference in T2DM individuals with sarcopenia.

## Materials and methods

### Study design and setting

A cross-sectional correlational study was conducted at the Center for Podiatry & Diabetic Foot Care and Research, the Department of Physiotherapy, and the Department of Endocrinology at Kasturba Hospital, Manipal, Karnataka, India.

### Ethics approval

The study was initiated once the Institutional Ethics Committee, Kasturba Hospital, Manipal (IEC1:426/2023), granted its clearance, and it was registered with the Clinical Trials Registry of India (CTRI/2024/02/062765). All willing participants were provided with complete details about the study, and written informed consent was obtained. We confirm that the research was conducted in accordance with relevant guidelines/regulations. As the research involved human participants, all the methods were performed in accordance with the Declaration of Helsinki.

### Inclusion and exclusion criteria

Participants who had a confirmed diagnosis of T2DM were recruited for the study. The inclusion criteria were: (1) age between 40 and 70 years, (2) Individuals diagnosed with T2DM and on medication. (3) Meeting sarcopenia diagnostic criteria according to the Asian Working Group on Sarcopenia (AWGS) 2019. The exclusion criteria were (1) any history of lower limb amputation and lower limb injury, (2) active lower limb ulcer, (3) Patients diagnosed with Cancer, and (4) the presence of any neurodegenerative disorder.

### Data collection

#### Anthropometric measurements

We collected the following patient data: age, sex, height, weight, duration of type 2 diabetes mellitus, random blood sugar values, glycated hemoglobin (HbA1C), and blood pressure. We assessed peripheral neuropathy using vibration perception threshold (VPT). A digital weighing scale and wall-mounted stadiometer were used to measure the subject’s height and weight. Skeletal mass index was assessed using Bioelectric Impedance analysis with an InBody 270.

#### Diagnostic criteria for sarcopenia

In our study, sarcopenia was diagnosed using the Asian Working Group on Sarcopenia Criteria (AWGS) 2019. Sarcopenia was defined by low appendicular skeletal muscle mass (ASM), low muscle strength, or low physical performance. Specifically, low muscle mass was defined as an appendicular skeletal mass index (ASMI) < 7.0 kg/m^2^ for men and < 5.7 kg/m^2^ for women (measured by bioelectrical impedance analysis); low muscle strength using hand grip strength was defined as < 28 kg for men and < 18 kg for women. Patients with both low muscle strength and low muscle mass were diagnosed with sarcopenia^17^.

### Procedure

The evaluation procedure was clearly explained to the participants, and permission to be assessed was obtained.

### Assessment of handgrip strength using Jamar handheld dynamometer

Participants were asked to sit in a relaxed position on a chair, with their elbows flexed to 90 degrees and their shoulders adducted, and to squeeze the Jamar handheld dynamometer (Jamar-5030J1; Patterson Medical Supply Inc., Chicago, IL) as hard as possible. Three trials were taken, and an average of three readings were noted. The assessment was performed only for the dominant hand^18^.

### Calf circumference measurement using inch tape

Using a non-stretchable inch tape, the participants were told to stand comfortably with their calf exposed. The therapist then took a horizontal measurement perpendicular to the leg’s long axis at the maximal circumference between the knee and ankle^16^.

### Skeletal mass index assessment using Inbody 270

The skeletal mass index was assessed using the BIA Inbody 270 (Inbody 270, India Pvt. Ltd), for which the participants were instructed to follow certain instructions, such as not to consume heavy meals for at least 2 h and water for at least 15 to 20 min before the test, refraining from consuming alcohol or caffeine 24 h beforehand, as these factors may influence test results. Participants were also instructed to remove ornaments and metal equipment that could interfere with the results. They were asked to stand on the machine’s foot-shaped electrodes until the weight was measured. The participant was asked to maintain a straight posture and look ahead, and was also asked to hold their hands slightly abducted from the body. The results were displayed on the monitor. Skeletal mass index was obtained directly from the displayed report from the InBody 270 device. The InBody 270 is a validated device for measuring SMI^19^.

#### Sample size

The required sample size was calculated using the sample size calculator for designing clinical research^20^.

$$\:N={\left[\frac{{z}_{\alpha\:}+{z}_{\beta\:}}{c}\right]}^{2}+3$$

Considering an expected correlation co-efficient of 0.5, Z_α_ = 1.96, Z_β =_ 0.84 and C = 0.54.

*N* = 29/ group, Total *N* = 58.

### Statistical analysis

The data were examined using Jamovi 2.3.28 software (Sydney, Australia). Demographic characteristics were analyzed using descriptive statistics. The Shapiro-Wilk test for normality was used to assess whether the data were normally distributed. Spearman’s correlation test was used to determine the correlation between calf circumference and skeletal mass index. In addition to the correlation analysis, a multiple linear regression analysis was performed to evaluate the independent association between calf circumference and skeletal muscle index, adjusting for age, sex, BMI, and duration of diabetes mellitus. We assessed the model assumptions using variance inflation factors (VIF) and normality testing of residuals. A p-value < 0.05 was chosen as the statistical significance level.

## Results

### Demographics and clinical characteristics

A total of 62 participants with type 2 diabetes mellitus met the full diagnostic criteria for sarcopenia according to the AWGS 2019 criteria. This required the presence of both a low appendicular skeletal muscle index and low hand grip strength. Among them, 29 were females, and 33 were males. The mean age of the female participants in this study was 58.24 ± 8.56 years, and that of the male participants was 61.09 ± 8.37 years. The demographic and clinical characteristics of the participants are shown in Table 1.

**Table 1:** Demographic and clinical characteristics of the participants.

Variables	Males (*n* = 33) Mean ± SD	Females (*n* = 29) Mean ± SD	Total (*n* = 62) Mean ± SD
Height (cm)	164.7 ± 6.54	152.27 ± 5.54	158.92 ± 8.72
Weight (kg)	62.12 ± 9.49	57.13 ± 7.35	59.79 ± 8.85
BMI (kg/m^2^)	22.92 ± 3.54	24.63 ± 2.88	23.72 ± 3.33
Systolic blood pressure (SBP) (mmHg)	137.47 ± 19.38	133.12 ± 14.9	135.45 ± 17.43
Diastolic blood pressure (DBP) (mmHg)	79.13 ± 10.43	77.38 ± 7.83	78.32 ± 9.28
Duration of type 2 diabetes mellitus (in years)	11.81 ± 9.52	9.39 ± 7.29	10.68 ± 8.57
HbA1c (%)	7.71 ± 1.2	7.47 ± 0.85	7.60 ± 1.05
Calf circumference (cm)	31.39 ± 2.08	30.40 ± 1.91	30.83 ± 2.03
Biceps circumference (cm)	24.39 ± 5.05	24.30 ± 5.15	24.4 ± 5.06
Hand grip strength (kg)	21.36 ± 6.90	13.0 ± 1.93	17.5 ± 6.65
Skeletal mass index (kg/m^2^)	6.59 ± 0.79	5.95 ± 0.72	6.29 ± 0.81
Vibration perception threshold (VPT) in Volts	25.2 ± 13.5	21.9 ± 11.9	23.9 ± 12.88

*SD* standard deviation, *BMI* body mass index, *HbA1c* glycated hemoglobin.

### Correlation between skeletal mass index and calf circumference

**Fig. 1:**
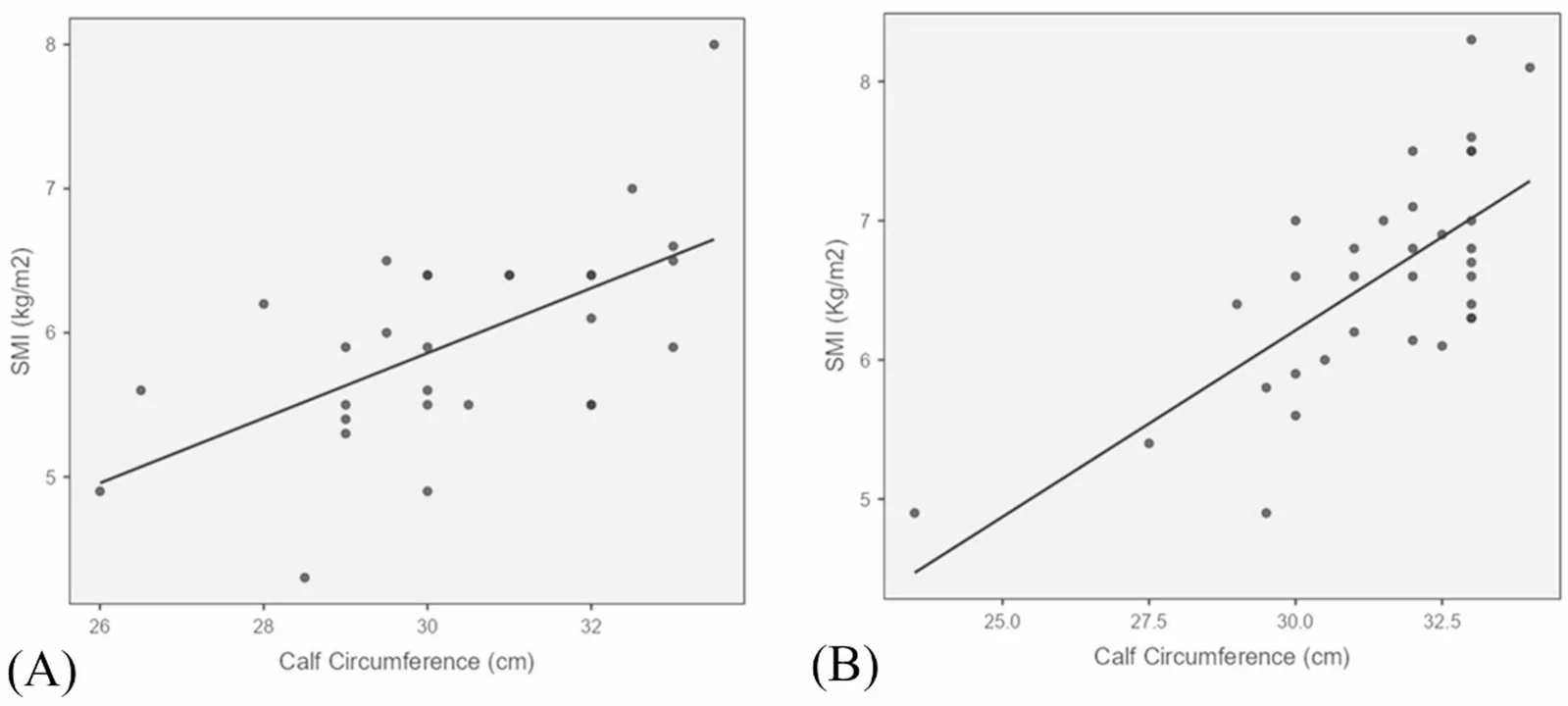
Scatter plot correlation between calf circumference and skeletal mass index; (**A**) females, (**B**) males.

The present study found a statistically significant, moderate, positive correlation between skeletal mass index and calf circumference for males and females (Table 2). The Spearman’s correlation coefficient between calf circumference and SMI for females was (*r* = 0.58, p-value < 0.001), and for males was (*r* = 0.62, p-value < 0.001). Figure 1.

**Table 2:** Correlation between skeletal mass index and calf circumference; skeletal mass index and HbA1c among females and males.

Variables	Males			Females		
	Mean ± SD	Correlation with SMI		Mean ± SD	Correlation with SMI	
		r-value	p-value		r-value	p-value
Calf circumference (cm)	31.39 ± 2.08	0.62	< 0.001	30.40 ± 1.91	0.58	< 0.001
HbA1c (%)	7.71 ± 1.2	-0.208	0.28	7.47 ± 0.85	0.26	0.21

*SD* standard deviation.

### Multivariable adjusted analysis

The multivariable linear regression model was statistically significant (R² = 0.556, adjusted R² = 0.515, F(5,54) = 13.547, *p* < 0.001), indicating that approximately 55.6% of the variance in SMI was explained by the model. After adjustment for age, sex, BMI, and duration of diabetes mellitus, calf circumference remained independently and positively associated with SMI (β = 0.183, 95% CI: 0.076–0.290, *p* = 0.001). This indicates that each 1 cm increase in calf circumference was associated with an increase of approximately 0.18 units in skeletal mass index Table 3. Male gender was also independently associated with higher skeletal mass index values compared to females (β = 0.597, 95% CI: 0.226–0.967, *p* = 0.002). However, age, BMI, and duration of diabetes mellitus were not significantly associated with SMI after adjustment Table 3.

**Table 3:** Multivariable linear regression analysis for predictors of skeletal muscle index (SMI).

Predictor	Estimate (β)	SE	95% CI Lower	95% CI Upper	t-value	*p*-value
Intercept	-0.611	1.387	-3.392	2.170	-0.441	0.661
Calf circumference	0.183	0.053	0.076	0.290	3.440	0.001
Age	-0.004	0.010	-0.025	0.016	-0.425	0.672
BMI	0.052	0.033	-0.014	0.119	1.581	0.120
Gender (male vs. female)	0.597	0.185	0.226	0.967	3.225	0.002*
Duration of diabetes mellitus	-0.005	0.010	-0.026	0.016	-0.468	0.642

### Regression assumption testing

Model diagnostics demonstrated acceptable normality of residuals (Shapiro–Wilk statistic = 0.978, *p* = 0.337). Multicollinearity assessment showed no evidence of significant collinearity among predictors, with all VIF values below 2.5. These findings indicate that the assumptions of linear regression were adequately met.

## Discussion

To the best of our knowledge, this is the first Indian study to report on skeletal mass index and calf muscle circumference in people with T2DM who also have sarcopenia. In the present study, we found a statistically significant, moderate positive correlation between calf circumference and skeletal mass index (*r* = 0.6). The finding indicates a clinically meaningful relationship, suggesting that calf circumference can serve as a simple, practical proxy for estimating skeletal muscle mass in T2DM participants with sarcopenia. A correlation of this magnitude is particularly valuable in clinical practice because calf circumference is simple, inexpensive, and requires minimal equipment, making it highly feasible for routine screening in resource-limited settings. Unlike more complex measures, it can be easily performed at the bedside or in community settings, potentially facilitating early identification of sarcopenia in high-risk T2DM populations.

Furthermore, multivariable linear regression analysis demonstrated that calf circumference remained independently associated with skeletal mass index even after adjusting for age, sex, BMI, and duration of diabetes mellitus. This suggests that calf circumference may serve as an independent marker of muscle mass in individuals with T2DM. Our findings support earlier research showing a substantial correlation between skeletal mass index and calf muscle circumference in sarcopenic individuals without T2DM. This could be due to decreased body mass index (BMI) in participants and decreased calf circumference, which is an early marker of sarcopenia in individuals with T2DM^13^. We found a decrease in calf muscle circumference, which can be correlated with reduced muscle mass in the elderly population. The regression model explained approximately 55.6% of the variance in skeletal mass index, indicating a moderate contribution of the included clinical variables. Calf circumference has been identified as a simple, non-invasive anthropometric measure that reflects peripheral muscle mass and can be useful for early screening of sarcopenia, particularly in low-resource settings^21^.

In this study, the average calf circumference for females was 30.4 ± 1.99 cm and 31.39 ± 2.08 cm for males. This study shows an interesting difference between males and females. Compared with males, females had lower calf circumference, suggesting they may be physically inactive, and lower skeletal mass index, placing them at higher risk for sarcopenia in the early stage. The study population consisted exclusively of individuals with low calf circumference, indicating a higher risk of sarcopenia.

A study conducted by Leonardo Pozza Santos et al. 2018 who used DEXA for the assessment of appendicular skeletal mass and calf circumference for the evaluation of sarcopenia, concluded that there exists a high correlation between skeletal mass index and calf circumference and suggested that calf muscle circumference can be used as a marker for muscle mass in early and middle-aged adults^22^. Similarly, Yun-Xia Zhu et al. (2020) found that decreased calf muscle circumference in diabetic patients may lead to frailty in older adults^23^.

When poor glycaemic control and insulin resistance exist, the loss of skeletal muscle mass in individuals with T2DM is disproportionately high. There is a direct correlation between poor glycaemic control and decreased muscle mass, strength, and overall physical performance^24^. In our study, patients with T2DM who had reduced muscle mass and sarcopenia were also had poor glycaemic control. In the current study, more than half of the participants had suboptimal glycaemic control (HbA1c ≥ 6.5)^25^. However, no significant association was observed between HbA1c and skeletal mass index. This suggests that glycaemic control alone may not adequately reflect muscle mass status, and that additional factors such as physical activity, nutritional status, and metabolic alterations may influence sarcopenia in T2DM^26^.

Peripheral neuropathy is a well-recognized complication of T2DM and has been identified as an independent risk factor for sarcopenia^27,28^. A study conducted by Qin Yang et al. (2020) examined the association between diabetic peripheral neuropathy (DPN) in T2DM and sarcopenia. They found that DPN is one of the independent risk factors for the development of sarcopenia in patients with T2DM^28^. Although peripheral neuropathy was assessed in the present study, its association with calf circumference and skeletal mass index was not analyzed.

As sarcopenia is prevalent in T2DM, it is necessary for early detection and management of patients. Early diagnosis of sarcopenia can be performed using simple, inexpensive tools, such as a non-stretchable inch tape to measure calf circumference, when facilities such as a bioelectric impedance analysis (BIA) device to estimate skeletal mass index are unavailable.

A decrease in the skeletal mass index is directly related to a change in the calf circumference. When calf circumference is reduced, a significant loss of muscle mass is also observed, as revealed by the skeletal mass index measured by BIA. Therefore, to reverse sarcopenia or to prevent deterioration of sarcopenia, an early exercise-based intervention is required.

### Limitations of the study

The present study has several limitations. The sample size was relatively small, which may limit the generalizability of the findings. Skeletal mass index was measured using BIA rather than gold-standard methods such as DEXA. Additionally, the detailed analysis or assessment of nutritional status was not performed. The study included participants with mild, moderate, and severe neuropathy (heterogeneous groups).

## Conclusion

There is a positive relationship between calf circumference and skeletal mass index among T2DM with sarcopenia. Based on the findings, the calf circumference measurement is a reliable, cost-effective, and time-saving method for screening for sarcopenia among T2DM patients. Therefore, this screening method can be incorporated in low-resource healthcare settings.

### Implications for clinical practice

In clinical practice, the assessment and diagnosis of sarcopenia in T2DM is time-consuming. The advanced methods used for diagnosing sarcopenia are DEXA, ultrasound Imaging, and many more diagnostic techniques that are not adequately available in different levels of healthcare settings. In these cases, we suggest assessing sarcopenia by measuring calf muscle circumference and SMI using the InBody 270 bioelectrical impedance analysis. Also, for community-level screening, measuring calf circumference and SMI are more convenient for screening sarcopenia.

## Data Availability

The datasets used and/or analyzed during the current study are available from the corresponding author on reasonable request.
